# Factors associated with improved survival following surgical treatment for metastatic prostate cancer in the spine: retrospective analysis of 29 patients in a single center

**DOI:** 10.1186/s12957-016-0961-y

**Published:** 2016-07-29

**Authors:** Tong Meng, Rui Chen, Nanzhe Zhong, Tianqi Fan, Bo Li, Huabin Yin, Zhenxi Li, Wang Zhou, Dianwen Song, Jianru Xiao

**Affiliations:** 1 Department of Bone Tumor Surgery, Changzheng Hospital, Second Military Medical University, 415 Fengyang Road, Shanghai, 200003 China; 2 Department of Urology, Shanghai Changhai Hospital, Second Military Medical University, 168 Changhai Road, Shanghai, China; 3 Department of Orthopedics, Shanghai Jiao Tong University, Shanghai First People’s Hospital, No. 100 Haining Road, Shanghai, 200080 China

**Keywords:** Prostate cancer, Spinal metastases, Surgical outcomes, Prognostic factors, Survival analysis

## Abstract

**Background:**

Prostate cancer (PCa) is very common and frequently metastasizes to the spine. However, PCa spinal metastases were rarely reported in the literature. In this study, the outcome of therapies and prognostic factors affecting surgical outcomes for patients with PCa spinal metastases are discussed to select the best candidates for aggressive surgical resection.

**Methods:**

All patients affected by the spinal metastatic PCa surgically treated at our spine tumor center were reviewed. Overall survival was analyzed from the time of spinal surgery. A univariate survival analysis and a multivariate Cox proportional hazard analysis to identify independent prognostic factors were carried out. The survival rate was estimated by the Kaplan-Meier method, and differences were analyzed by the log-rank test. Factors with *P* values of 0.1 or less were subjected to multivariate analysis for survival rate by multivariate Cox proportional hazard analysis.

**Results:**

A total of 31 consecutive patients were identified. Of these, 29 underwent surgical resection. The median survival time of all patients after their spinal surgery was 44.0 months. Visceral metastases, revised Tokuhashi scores (0–8/9–11/12–15), Tomita scores (7–10/2–6), hormone status, and bisphosphonate treatment were suggested as the potential prognostic factors through univariate analysis. As they were submitted to the multivariate Cox regression model, visceral metastases and Tomita score were found as independent prognostic factors.

**Conclusions:**

Patients without visceral metastases and a Tomita score no more than 6 are favorable prognostic factors for PCa metastases in the mobile spine.

## Background

Prostate cancer (PCa) is the second leading cause of cancer-related deaths worldwide currently, with an estimated 238,590 new diagnoses and approximately 29,720 related deaths in the USA in 2013 alone [1]. The morbidity has increased substantially recently in China, with an estimated incidence of 10/100,000 in 2010 compared with 1.71/100,000 in 1993 [2]. Although most patients respond to surgery or radiation well, more than 80 % of patients with advanced prostate cancer develop bone metastases later in their lives, with the spine being the most commonly affected site [3]. The incidence of skeletal-related events, such as pathologic skeletal fractures, neurologic deficit, and intractable pain, secondary to prostate metastatic spinal lesions, raises considerable high risks on both patients’ quality of life and their health-care costs as with other osteolytic metastases [4].

In most cases, bone pain in newly diagnosed metastatic PCa is responsive to lowering testosterone to castrate levels (termed “castration sensitive”), and mild spinal cord compression responds well to external beam radiation therapy [5]. However, vertebral compression fractures and segmental instability, along with progressive neurologic deficits and para- or tetraplegia, collectively make operative intervention mandatory [6]. Several studies have shown that surgical treatment options are both important and effective for these patients [7–9]. However, all surgical procedures of metastatic spinal disease remain palliative by definition, and thus, clinicians are still hesitant to choose surgery when faced with vertebral metastases.

Therefore, it is necessary to perform a systematic analysis of all patients operated for metastatic PCa in our hospital to evaluate the life expectancy and prognosis which may help to select the best candidates for aggressive surgical resection.

## Methods

A retrospective review was performed on all patients treated at our spine tumor center from April 2002 to March 2012 for surgical treatment of spinal metastases. During this period, 31 consecutive patients were histologically confirmed with PCa with metastases to the spine. All of them were evaluated by multidisciplinary consultants to provide therapeutic strategy, and the indications for surgical intervention were rapidly progressive neurological deterioration and pathologic skeletal fracture with the life expectancy of more than 6 months. Permission from the hospital ethics committee was obtained before commencing this study, and an informed consent was required from all patients or their legal guardians.

Of the 31 patients, 2 patients did not undergo surgical treatment. One patient was due to the life expectancy of less than 6 months, and the other is because osteoblastic lesions did not destroy the stabilization of the spine. In this regard, a total of 29 patients were included in this study. Medical records of all patients are retrospectively retrieved for clinical and operative reports, radiographic images, and pathology reports. Data collected regarding primary PCa included age at initial diagnosis, Gleason score, preoperative serum PSA, and treatment modalities, including surgery, radiation, and/or chemotherapy/hormonal therapy. Data collected regarding the spinal metastases included the following: age at diagnosis of spinal metastases; Karnofsky performance score (KPS) which was recorded just before surgery; presenting signs and symptoms, including neurological function quantified by the Frankel grading system; visceral metastases; location; local treatment with cisplatin or methotrexate; bisphosphonate treatment; adjuvant therapy (radiotherapy and/or chemotherapy); and clinical scores, including Tomita scores [10], revised Tokuhashi scores [11], and Crnalic prostate scores [8].

Before surgery, patients were examined with X-rays for the affected spinal segments, computed tomography (CT) of the involved vertebra, and magnetic resonance (MR) imaging focused on the affected spinal segment to monitor the extensiveness of the metastatic process and spinal canal involvement. A systemic search for other metastases was performed including abdominal sonography and chest X-ray to detect pulmonary metastases. A selective ^18^F-fludeoxyglucose positron emission tomography is performed to increase sensitivity and specificity to detect other metastatic lesions according to patients’ informed decision.

Postoperative survival as a function of time was defined as the interval from the date of spine surgery to death or until July 2014 for alive patients and expressed using the Kaplan-Meier method. The log-rank test was used in univariate survival analysis to identify independent variables that could predict prognosis. Clinical experience and statistical analysis were used to decide whether continuous variables should be categorized. Factors with *P* values ≤0.10 were subjected to a multivariate model for survival rate by multivariate Cox proportional hazard analysis. *P* values ≤0.05 were considered statistically significant. All statistical calculations were performed by PASW Statistics, version 19.0.

## Results

The study population included 29 consecutive patients, with a median age of 71 (range 59–83) years. A summary of all variables tested by log-rank test is provided in Table 1. Fifteen out of 29 patients were with a mean duration of 21.5 (range 1–83) months from diagnosis of PCa to spinal metastases, and the spinal metastases were the initial manifestation of PCa in the remaining 14 patients. The diagnosis of spinal metastases was upon initial examinations and pathological examination at our institution. There were 20 hormone-naïve patients including 14 patients with spinal metastases as the initial manifestation of PCa and 6 patients who previously underwent prostatectomy and did not receive hormonal therapy.

**Table 1:** Univariate analysis of the prognostic factors affecting survival

Factor	Number	*P* (log-rank test)
Age		
<65/≥65	8/21	0.128
Location		
Cervical/thoracic/lumbar/sacral	5/11/12/1	0.306
Junctional/mobile spine/semirigid/rigid	8/13/8/0	0.464
Karnofsky performance status		
100–80/70–50/40–10	3/17/9	0.071
100–80/70–60/50–10	1/14/14	0.364
Visceral metastases		
Present/not present	7/22	0.016*
Number of vertebral metastases		
1/2/≥3	9/1/19	0.505
Number of extraspinal metastases		
0/1–2/≥3	19/2/8	0.920
Preoperative Frankel score		
A–C (not ambulatory)/D–E (ambulatory)	19/10	0.713
Revised Tokuhashi scores		
0–8/9–11/12–15	7/16/6	0.048*
Tomita scores		
7–10/2–6	6/23	0.084*
Crnalic prostate scores		
0–1/2–4/5–6	7/20/2	0.133
PSA		
<100/≥100	13/16	0.843
Hormone status 1		
Hormone-naïve/hormone-refractory	20/9	0.024*
Hormone status 2		
Hormone-naïve 1/hormone-naïve 2/hormone-refractory	16/4/9	0.109
Bisphosphonate treatment		
Yes/no	16/13	0.011*
Urinary and bowel continence		
Yes/no	24/5	0.900
History of PCa		
Yes/no	15/14	0.386

Hormone-naïve 1 means patients with spinal metastases as the initial manifestation of PCa. Hormone-naïve 2 means patients who previously underwent prostatectomy and did not receive hormonal therapy

*P value less than 0.1 for the multivariate analysis

Most of our patients (27/29) have a similar symptom that localized pain, along with radiating pain, was first to occur and progressive neurological defects occurred afterwards. Acute pathologic skeletal fracture was the first symptom of the remaining 2 patients. Symptoms of night pain, muscle weakness, and even paraplegia were the most common reasons for patients to see doctors. After surgery, localized back pain disappeared in all patients and Frankel scores at postoperative 6 months were significantly improved by at least one level compared with the preoperative scores (*P* < 0.05) (Table 2).

**Table 2:** Frankel grade during follow-up

Frankel grade	Preoperative	Postoperative (1 month)	Postoperative (6 months)^a^
E	2	3	3
D	8	12	16
C	13	10	6
B	4	4	3
A	2	0	0

^a^This patient died 1 month after surgery due to postoperative cerebrospinal fluid infection

For patients who had more than one metastatic lesion of the spine, operations were performed depending on the degree of the spinal compression. Other osteolytic lesions which did not lead to spinal compression were treated by percutaneous vertebroplasty (PVP). There were no specific differences in surgical management specific to PCa compared to the metastases from other tumors. Curettage and reconstruction of the spine were performed in all cases. In thoracic and lumbar metastases, dorsal spinal decompression and fixation was the standard surgical technique. Fixation was achieved by transpedicular screws and rods in 24 patients. Reconstruction was achieved by a titanium mesh cage placement in 10 patients and by cement-wire construct placement in 14 patients. In cervical metastases, a ventral/dorsal/combined (1/3/1) approach of decompression was performed, with stable-angle plate osteosynthesis in the ventral approach, transpedicular screws in the dorsal approach, and both in the combined approach.

Bisphosphonate was used after surgery in 16 cases, with incadronate disodium at a dose of 10 mg in 500-ml normal saline intravenous infusion 2 h once a month for postoperation 2 years. Thirty-one percent (*n* = 9) of the sample sustained one or more postoperative complications, including pulmonary infection, incision infection, cerebrospinal fluid infection, and lower extremity deep venous thrombosis. One patient died within the first 30 days of surgery because of postoperative cerebrospinal fluid infection. By the end of the study period, 18 patients were still alive. The median survival time of all patients after their spinal surgery was 44 (range 1–80) months, and the median survival time for survivors was 26 (range 14–80) months. The 1-year overall survival was 89.7 %, 2-year overall survival was 74.4 %, and 5-year overall survival was 19.7 %.

### Univariate analysis of prognostic factors

The univariate analysis of the prognostic factors affecting survival time is shown in Table 1. The factors such as visceral metastases, revised Tokuhashi scores, hormone status, and bisphosphonate treatment showed the significant effect for the patients’ overall survival in the univariate analysis. In our series, patients without visceral metastases had a better survival rate (*P* = 0.016). Among the possible prognostic factors, the difference of overall survival rate between patients with better revised Tokuhashi scores and the worse ones was statistically significant (*P* = 0.048). Patients with hormone-naïve disease earned a longer life than those with hormone-refractory disease (*P* = 0.049), and the use of bisphosphonate treatment also effected the survival period (*P* = 0.011). No significant difference was found in other factors of age, tumor location, KPS, number of vertebral metastases, number of extraspinal metastases, hormone status 2, Tomita scores, Crnalic prostate scores, preoperative Frankel score, preoperative PSA, urinary and bowel continence, and history of PCa.

### Multivariate analysis of prognostic factors

**Fig. 1:**
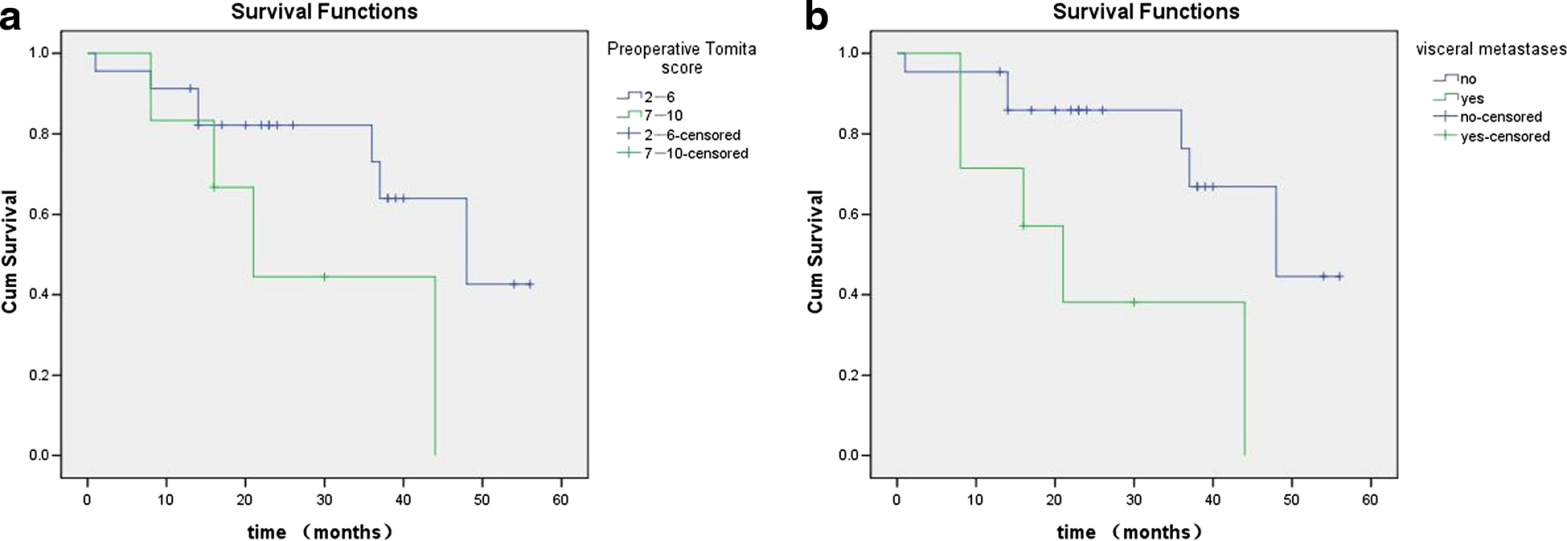
Kaplan-Meier curves of OS for **a** preoperative Tomita score and **b** visceral metastases

Univariate analysis suggested that the potential prognostic factors were visceral metastases, revised Tokuhashi scores (0–8/9–11/12–15), Tomita scores (7–10/2–6), hormone status, and bisphosphonate treatment. They were submitted to the multivariate Cox regression model for analysis of overall survivals. The risk of death was significantly decreased in patients with a Tomita score of 6 or less compared with patients with a Tomita score of 7 or more. The hazard ratio was 0.008 (95 % confidence interval (CI) 0.000–0.743) for survival (*P* = 0.037). The risk of death was significantly decreased in patients without visceral metastases compared with patients with visceral metastases. The hazard ratio was 199.232 (95 % CI 2.615–15,180.426) for survival (*P* = 0.017). The Kaplan-Meier curves of survival for Tomita score and visceral metastases are shown in Fig. 1. The result of multivariate analysis showed that revised Tokuhashi scores, the method of hormonal therapy, hormone status, and bisphosphonate treatment were not independent prognostic factors for overall survival. Details are listed in Table 3.

**Table 3:** Multivariate analysis of the prognostic factors affecting survival

Factor	HR (95 % CI)	*P*
Tomita scores	0.008 (0.000–0.743)	0.037*
Visceral metastases	199.232 (2.615–15,180.426)	0.017*
Revised Tokuhashi scores		0.294
Revised Tokuhashi scores (1)	1.271 (0.039–41.351)	0.893
Revised Tokuhashi scores (2)	0.235 (0.028–1.967)	0.182
Hormone status	0.532 (0.062–4.596)	0.566
Bisphosphonate treatment	0.093 (0.005–1.631)	0.104

*HR* hazard ratio, *CI* confidence interval

*P values less than 0.05 were considered statistically significant

## Discussion

More comprehensive testing methods for the early diagnosis of PCa and more effective treatments, such as transurethral resection of the prostate (TURP) or androgen deprivation therapy, have led to the favorable long-term survival of patients with PCa. Nowadays, only a few series in the literature focus on the therapeutic outcomes and prognostic factor related to metastatic spinal PCa [7–9, 12, 13] and there is no report from developing countries. China’s, as the biggest developing country, views on disease and the relative backward economic level result in some differences about the patient composition of metastatic PCa from those in developed countries. So, we presented this study to add more information on the treatments of spinal metastases secondary to PCa.

As spinal metastases from PCa are radiosensitive [14], whether to apply a surgical intervention to metastatic disease is always controversial. Surgical treatment for metastatic spinal disease always strive for the maximum palliative effect, including relieving pain and restoring the function and stability of the spine, with a minimum of operative mortality [7, 9, 15]. In our study, localized pain and radiating pain were presented in all patients and disappeared in all these patients after surgery. Thirteen of 19 non-ambulatory patients regained their ambulatory ability, but 1 patient experienced symptom deterioration because of other level compressions. It means that the improvement of ambulatory capability could be maintained until other spinal cord compressions occurred. Therefore, the benefit of surgical procedures on patients with paraplegia caused by PCa metastases is apparent not only to patients themselves but also to society, because many patients received restoration of their ability to care for themselves. However, 2 patients who presented with complete paralysis preoperation for more than 2 weeks did not regain their ambulatory ability. Therefore, it is limited to improve the degree of neurological function for patients presented with complete paralysis.

### Age and Karnofsky performance status

The impact of age and preoperative KPS on predicting survival after surgery for PCa spinal metastases is still contradictory. Crnalic et al. [8] reported KPS as a strong predictor of survival, and Ju et al. [9] reported that age younger than 65 years was associated with increased risk of postoperative complications, whereas both of those were not related to survival in our study according to the univariate analysis. In our opinion, age and KPS, considered as the general condition, may be controlled properly by an increased monitored anesthesia care, surgical experience, and surgical techniques [16]. In the present study, metastases occurred in patients aged 71 years old (range 59–83), which was older than those presented previously [7–9, 12, 13]. Furthermore, patients presented with severely impaired KPS (≤50) were more than other studies [7–9, 12, 13].

The relatively old patient age and low KPS in our study may be attributed to the size of the cohort, the underdeveloped testing methods in China, and the negative attitude of Chinese people to seek medical advice. In addition, insufficient health insurance in China may be another reason. All these would count for late diagnosis and reduce patients’ survival. In facing the patients with old age and/or low KPS, the most important thing is to evaluate their tolerance to surgical treatment. Our experience is that old age or low KPS is not the surgical contraindication. If the patient can well tolerate the surgical operation and anesthesia, there was no difference between patients with low KPS and those with high KPS.

### Metastatic burden

The bone and visceral spread and the extent and number of bone metastases have been accepted as predictive factors for the patient’s survival [8, 12]. We found an association between the presence of visceral metastases and decreased survival according to multivariate analysis. The maximum frequency of spine involvement occurred in smaller prostate tumors (4–6 cm) as compared with the maximum spread to the lung (6–8 cm) and liver (>8 cm) [3]. Therefore, visceral metastases, presented with heavier metastatic burden than spine metastases, developed in the late stage of PCa. We agree with Crnalic et al.’s opinion [8] that the presence of visceral metastases will further depress their tolerance to surgery and make them susceptible to complications, consequently having a potentially negative effect on survival.

In the present study, metastatic lesions usually are presented multiple (20 cases), which is in accordance with previous studies [14, 17]. Furthermore, 3 of the remaining 9 cases were treated before 2006, in which year we did not have PET-CT and would not find other metastases. Although the number of vertebral metastases was not an independent factor for overall survival according to the univariate analysis, the presence of multiple metastases may significantly influence surgical planning regarding the level of reconstruction [9].

### Androgen deprivation therapy

Androgen deprivation therapy is the gold standard for advanced PCa and can prolong the survival of men with advanced PCa. Hormonal treatment can be applied in receptor-positive prostate cancer patients [18]. The result of our univariate analysis revealed that patients with hormone-naïve disease had a better prognosis compared to hormone-refractory ones, which is in accordance with previous studies [7, 8]. However, multivariate analysis revealed that it was not an independent prognostic factor. It may be attributed to the small size of the cohort, whereas, in our opinion, patients with hormone-naïve disease present at an earlier phase of metastatic PCa progression and are usually in good general condition. Accordingly, we still regard patients with hormone-naïve disease as potentially suitable candidates for surgery [9].

### Bisphosphonates

Osteolytic and osteoblastic lesions were found in most radiographic images and operative reports in this study, which is in accordance with Michaelson and Smith’s study [19]. The bisphosphonates were used to inhibit osteoclast activity by cellular mechanisms that affect osteoclast attachment, differentiation, and survival and to reduce osteoclast activity indirectly through effects on osteoblasts. In our department, bisphosphonate had not been used until 2007. In this study, 16 patients had received regular bisphosphonate treatment after surgery, and their survival were longer than others according to the univariate analysis. This makes our study being the first one to investigate postoperative effect of bisphosphonates in prolonging the survival time of the patients with spinal metastases secondary to PCa [20–22]. Moreover, to our knowledge, the antiangiogenic effects and antitumoral activity of bisphosphonates can also contribute to restrain the growth of tumors [23]. Therefore, bisphosphonate treatment has been an integral component of the current treatment concept of spinal metastases in our hospital and, importantly, the use of bisphosphonates must be balanced with their complications [24].

### Prognosis scores

We evaluated the prognosis scores of Tomita, revised Tokuhashi, and Crnalic for life expectancy, and the results suggested that the Tomita scoring system indicated prognosis properly according to multivariate analysis. Nowadays, various scoring systems have been proposed to estimate which patients with spinal metastases would benefit most from surgery, and the histological type of primary tumor was placed on an important position. Moreover, Crnalic and his colleagues [8] presented a score for predicting survival particular for metastatic PCa. However, we agree with Enkaoua et al. [25] who thought it is a drawback to apply prognosis scores completely in an acute clinical situation, and to each retrospective study, it was challenging to assess all parameters of the score accurately, on account of the shortage of characteristics. Thus, we suggest that scoring systems may help but cannot be used as the only criteria in decision-making a surgery.

Although our study is a detailed analysis carried out for spinal metastases from PCa and has described surgical outcomes in a consecutive series, it has some limitations. First, it is a retrospective investigation which results in a certain degree of missing records and lack of a control group. Second, the patients are limited to only metastatic spinal PCa at a single spine tumor center within a restricted time period, and the incidence in China is lower than that of developed countries. Therefore, the sample size of our study is not big. While recognizing these limitations, it is hoped that this study may serve as a baseline for future prospective investigations. Our future studies will aim to enroll larger groups of PCa-only patients in clinical trials in a prospective research design.

## Conclusions

With the significant improvements in surgical experiences and expertise in dealing with different metastases problems, we suppose age and KPS are not the key points to impact postoperative survival. Patients without visceral metastases and a Tomita score no more than 6 are favorable prognostic factors for PCa metastases in the mobile spine.

## Abbreviations

CT, computed tomography; KPS, Karnofsky performance status; MR, magnetic resonance; PVP, percutaneous vertebroplasty; PCa, prostate cancer; TURP, transurethral resection of prostate
